# Existing Meditation and Breathing Devices for Stress Reduction and Their Incorporated Stimuli: A Systematic Literature Review and Competition Analysis

**DOI:** 10.1016/j.mcpdig.2023.06.008

**Published:** 2023-08-08

**Authors:** Elisabeth Honinx, Stefanie Broes, Bente Roekaerts, Isabelle Huys, Rosanne Janssens

**Affiliations:** a Moonbird B.V., Brussels, Belgium; b Department of Pharmaceutical and Pharmacological Sciences, KU Leuven, Leuven, Belgium

## Abstract

To identify, evaluate, and summarize existing meditation and breathing devices for stress reduction and their characteristics (stimuli), we searched PubMed and EMBASE for studies published from January 1, 1980, to December 30, 2021. Full-text articles that presented devices that support meditation and breathing guidance for stress reduction were included. We excluded articles covering study protocols. In addition, a competitor analysis was conducted to identify and evaluate the devices, their name, manufacturer, target group, function, and incorporated stimuli were contracted. The scientific literature identified 367 studies, of which 20 (describing 18 devices and 32 stimuli) were included. The competition analysis identified 66 devices incorporating 126 stimuli. After exclusion of duplicates, this resulted in 46 different types of devices incorporating 24 different types of stimuli. They were organized into 7 device categories (attachable to the head, huggable devices, handheld devices, eye masks, mouth-controlled devices, toys, and wearables) and 14 stimuli categories (perceptible vibrations, imperceptible vibrations, movement, temperature, pressure, texture, light or darkness, abstract visuals, concrete visuals, music, calming sounds, tones, voice guidance, and aromas). This review provides an overview of the different meditation and breathing devices for stress reduction. The current overview will inform a subsequent preference and effectiveness study of breathing devices that aims to gain more knowledge on the topic to increase device uptake and developmental success.

**Article Highlights**

• Currently, there are a variety of tools and devices available to help people regulate their breathing for stress reduction.
• There is a lack of evidence-based information and development of these tools and most devices are co-developed without target users.
• A thorough understanding of users’ preferences toward these devices’ characteristics becomes paramount to optimize design, user experience, and success rates.
• This overview of existing devices and their stimuli, which will be used to inform subsequent patient-preference studies and inform the development of new devices for stress reduction.

According to the WHO, stress can be defined as any type of change that causes physical, emotional, or psychological strain. It is the body’s response to anything that requires attention or action.^1^ Stress is considered one of today’s major threats to health.^2^ In our fast-paced society, stress and stress-related disorders, such as anxiety or sleeping problems, are highly prevalent and continue to increase; more than 1 in 3 people indicate they experience stress (∼35%).^3^, ^4^, ^5^ Stress plays a major role in the development of mental and physical disorders and is also associated with a greater risk of developing chronic disease (eg, cardiovascular disease, diabetes, and obesity).^6^ The stress response is regulated by the sympathetic nervous system (SNS), the branch of the autonomic nervous system that prepares the body for action.^7^ It causes the heart rate, blood pressure, and adrenaline levels to rise. This response is referred to as the fight or flight response, which stems from a carefully orchestrated evolutionary mechanism that mobilizes physical resources to help humans cope with life-threatening situations.^8^ Stress, is thus a normal reaction. It is a much-needed reaction to perform a variety of daily activities.^9^ Unfortunately, the body can also overreact to stressors that are not life-threatening such as relationships, workload, traffic jams. In addition, chronic stress leads to an imbalance in the body, which presents as increased activity in the sympathetic nervous system and decreased activity in the other branch of the autonomic nervous system, the parasympathetic nervous system, that regulates the relaxation response.^9^ Chronic stress has a negative effect on the mental and physical health and results in complaints like physical pain, anxiety, trouble sleeping, and loss of focus.^10^

Stress-related disorders are traditionally treated with medication. However, this has several disadvantages. One is that medication (eg, sleep medication) is often accompanied by negative adverse effects (eg daytime sleepiness, dizziness, gastrointestinal complaints, and balance disorders).^11^ Medication can also be addictive or result in tolerance, with potentially long-term negative physical and mental effects.^12^ These disadvantages lead to an increased need for nonpharmacological approaches. Among them, meditation, relaxation therapy, and mindfulness have recently been put forward as effective alternative treatments for stress-related problems.^13^ Most of these include breathing exercises as a central component. In fact, breathing exercises are used as effective treatments for stress, anxiety disorders, and sleeping problems.^14^, ^15^, ^16^

One breathing technique involves conscious, controlled slow breathing, in which practitioners slow down their breathing rate to a frequency lower than the resting breathing pace.^17^ It consists of controlled inhalations and exhalations, within the range of 4-10 breaths/min.^18^ Research has shown that practicing slow-paced breathing triggers the parasympathetic nervous system and increases heart rate variability (HRV), a marker of physiologic resilience.^19^, ^20^, ^21^, ^22^ Because slow breathing increases HRV, it therefore enhances autonomic reactivity to physical and mental stress, both short-term and long-term.^23^, ^24^, ^25^ By contrast to uncontrolled fast breathing, which is generally linked to anxiety and stress, slow-paced breathing techniques have been associated with relaxation and wellbeing.^23^^,^^26^ They are therefore considered an efficient and evidence-based approach to treating stress.^27^

Breathing exercises are often self-guided, which requires focus, patience, and commitment.^28^ People often struggle to sustain motivation, fail to keep focus on the breath, or lack sufficient self-awareness. All of this hinders continuous practice or may lead to frustration. Research indicates that externally-paced (guided) breathing is more effective in improving HRV than self-paced breathing.^29^ Guided breathing enhances engagement, provides assistance, and ensures that users perform exercises correctly.^30^^,^^31^

Currently, there are a variety of tools and devices available to help people regulate their breathing. These tools present breathing guidance by sensory stimulation, meaning that one or more of the senses are stimulated using visual, auditory, tactile, olfactory, or gustatory stimuli.^32^ Users are supposed to match their breathing with the rhythm provided by these stimuli. The advantage of using sensory stimuli as guidance is not only that they are easier to follow and are more effective than self-guided breathing, but stimulation itself can elicit a relaxation response, eg calming music or comforting aromas.^33^ Indeed, sensory stimulation (in particular tactile stimulation) increases the production of oxytocin and dopamine, which reduce stress and anxiety and induce wellbeing.^34^ This means that both slow breathing and sensory stimulation induce relaxation, which they reinforce each other’s effect.

Many relaxation devices using sensory stimuli are commercialized. Some are substantiated by scientific studies but the most are merely on the basis of intuition, on the idea of what feels good or what sells best.^28^ Despite the potential and promising effects of these meditation and breathing devices, there is a lack of evidence-based information and development of these tools. Furthermore, most devices are co-developed without target users, which leads to uncertainty about how these devices are received by them.^28^ Given the heterogeneity in views between users and the scarcity of evidence on stimulus preference, no device can be considered superior yet. Preliminary research indicates that people show a preference regarding which solutions they feel are most important in relieving stress, including specific types of devices and sensory stimuli, and suggests that these different devices and stimuli effect the physiological and psychological stress response in different ways.^28^^,^^35^ This research is, however, scarce and therefore still nonconclusive. Evidence from user preference studies is thus particularly valuable in these situations.^36^

Because target users have valuable insights toward necessary features of stress-relieving tools, the effectiveness and success of these tools depend on how well their preferences and needs are met. Using knowledge about user preferences and needs in the development of new breathing devices is also advantageous for the users themselves: more personalized support is possible, and greater user satisfaction is achieved together with more adherence to the product.^37^

A profound scientific understanding of the different sensory stimuli used in devices for stress reduction and the preferences toward them is a prerequisite for providing optimal guidance. The aim of this systematic literature review is to identify, evaluate, and summarize existing meditation and breathing guidance devices for stress reduction and the different sensory stimuli used within them. Findings from this review will be useful in informing subsequent patient-preference studies that assess patients’ preferences toward these characteristics and inform the development of new devices for stress reduction.

## Methods

This study was performed as part of the Flanders innovation and entrepreneurship (VLAIO) innovation project PreRest, funded by the Flanders Government (grant no.HBC.2021.0232). It was conducted in collaboration with Moonbird—the grant recipient—and KU Leuven.^38^ Moonbird is a Belgian healthtech startup (2019) that created a tactile breath guidance device for stress, anxiety, and sleep. The funder of the study had no role in study design, data collection, data analysis, data interpretation, or writing of the report.

A systematic literature review of scientific and gray literature was performed by both an extensive literature search (KU Leuven) and a competitor analysis. For the search, identification, and screening of studies for inclusion in the review, the PRISMA guidelines were followed.^39^ (PRISMA guidelines, Supplementary material, available online at https://www.mcpdigitalhealth.org/)

### I Search Strategy and Selection of Scientific Literature

PubMed and EMBASE were searched to retrieve literature on devices and sensory stimuli supporting breathing guidance for stress reduction and meditation. The search term was constructed using free text words (in title or abstract) and MeSH terms that were synonyms for concepts related to stress, breathing, device, and treatment (Supplemental Appendix 1, available online at https://www.mcpdigitalhealth.org/). The articles identified were screened by 2 researchers according to the following inclusion criteria: (1) studies that presented tools or devices that support breathing guidance for stress reduction and meditation; (2) studies that focused on human beings; (3) studies published from January 1, 1980, to December 30, 2021; (4) studies in English; and (5) full-text articles.

Articles covering study protocols or covering devices that were already included using other databases were excluded. The manual review was performed in 2 phases. Abstracts and titles were screened to identify those relevant to the research question. When limited information was available to determine eligibility, full articles were screened. Relevant articles were then selected by cross-examining the full-text articles.

#### Data Collection and Extraction Process

A data extraction form was developed on the basis of earlier patient preferences research.^40^ In this form, different aspects of the devices were described, such as name, manufacturer, target group, function, and the incorporated stimuli (Supplemental Appendix 2a, available online at https://www.mcpdigitalhealth.org/). One reviewer extracted the data, which was then checked by a second reviewer. Disagreements in data extraction were resolved through discussion with the co-authors.

### II Search Strategy for Competitive Analysis

For the competitive analysis, a step-by-step methodology, grounded on previous research,^41^, ^42^, ^43^, ^44^, ^45^, ^46^ was used to identify and classify alternative products to the Moonbird device. The primary criterion for a competing product was defined as any product or service that can be used by the target group as an alternative to the Moonbird device, that is, both a solution for stress and a meditation aid with breathing guidance.

#### Identifying Competing Products

The identification process involved conducting extensive online research using key terms such as breathwork, along with reviewing books and reports. The resulting products were categorized into 2 groups: Direct competitors—these are technological physical products offering breathing guidance such as activity trackers; and Indirect competitors—these encompass both technological products that do not measure or display a breathing rate and nontechnological products like meditation books. An effort was also made to anticipate future competitors by scanning crowdfunding platforms and patent applications. In particular, we were looking for products or devices being developed by companies already operating in this sector.

#### Data Analysis

For each identified product, quantitative data were collected and evaluated, such as product name, manufacturer, target user group, functional features, incorporated stimuli, and a quantified strengths and weaknesses analysis on the basis of set parameters such as user reviews, pricing, market presence, etc. This comprehensive approach ensured a robust and quantifiable competitive landscape.

## Results

**Figure 1:**
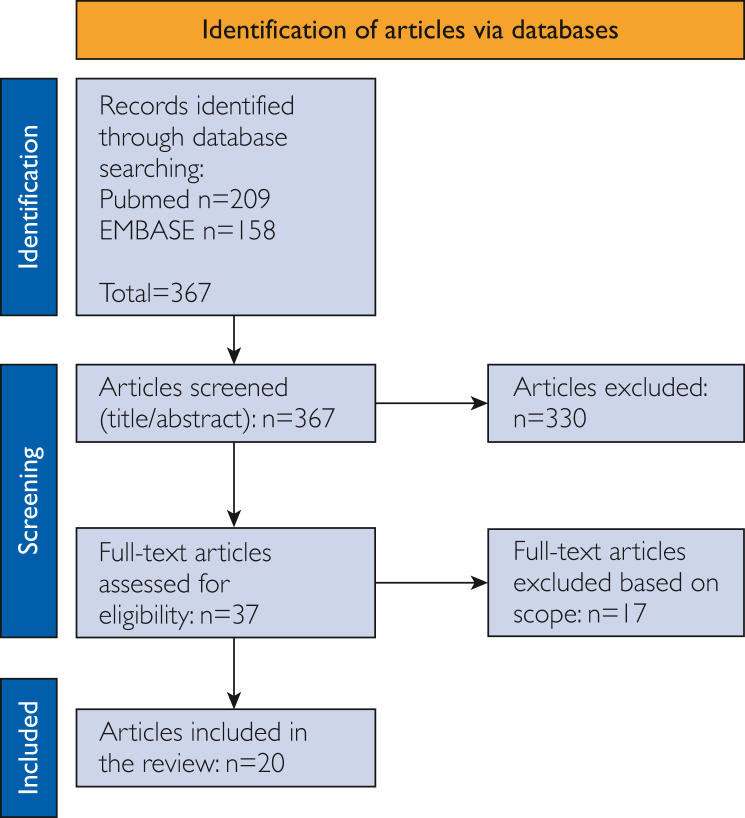
PRISMA flowchart of search, identification, and screening of studies for inclusion in the review. PRISMA indicates Preferred Reporting Items for Systematic Reviews and Meta-Analyse.

The flow of the systematic literature search is shown in Fig 1. Of the 367 articles detected in total, eventually 20 unique studies were identified that met the inclusion or exclusion criteria.^31^^,^^32^^,^^47^, ^48^, ^49^, ^50^, ^51^, ^52^, ^53^, ^54^, ^55^, ^56^, ^57^, ^58^, ^59^, ^60^, ^61^, ^62^, ^63^, ^64^ All 20 studies in the scientific literature reported on adult participants. Ten of them included patients (all types of conditions), the other 10 included participants from the (healthy) general population. Two studies used a cross-sectional design, 18 were prospective cohorts; 15 were interventional; 4 were experimental; and 1 consisted of a systematic literature review. The search yielded 18 devices that used 32 guiding and feedback stimuli. Using the competitive analysis 66 devices were found, incorporating 126 stimuli (Supplemental Appendix 2a). In total, after the exclusion of duplicates, 46 unique types of devices incorporating 24 unique types of stimuli were included (Supplemental Appendix 2b, available online at https://www.mcpdigitalhealth.org/).

A categorization of all included devices and stimuli was developed through discussion with the co-authors using a mixed bottom-up and top-down approach. First, we introduced a category for the type of products or devices (eg, wearables). Second, their sensory stimuli were categorized according to sense (tactile, visual, auditory, and olfactory). Gustatory senses were excluded because no guidance tools make use of this sensory stimulation. Third, these stimuli were divided into guiding stimuli—that indicate how to breathe—and feedback stimuli—indicating how well the user is breathing and what the effect of the breathing exercise is on his or her body.^65^^,^^66^ Finally, we reported on the feasibility, acceptability, usability, preferences, and effectiveness of the devices as described in the scientific literature.

### Types of Devices

The devices were organized into 7 categories regarding type of device: Attachable to the head, huggable devices, handheld, eye masks, mouth-controlled devices, toys, and wearables (Box 1, Supplemental Appendix 2b)*.*

**Box 1:**
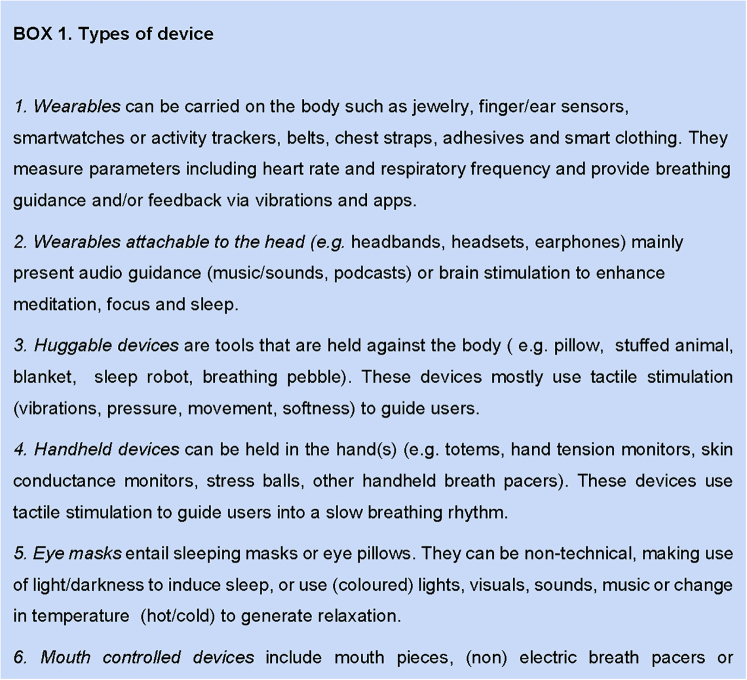
Types of devices.

Some devices were not categorized under types of devices but were integrated under types of stimuli. These devices do not need to be operated in a particular way to provide stimuli; they present stimuli on their own, as mentioned in the following: (1) visual stimuli (applications, projectors, game, or biofeedback devices); (2) auditory stimuli (applications, games, sound machines, speaker tools, and auditory interventions); (3) olfactory stimuli (aromatherapy); and (4) products that simultaneously provide visual, tactile or auditory breathing guidance or feedback (eg, companion robots).

### Types of Stimuli

The types of stimuli were divided into 14 categories (Supplemental Appendix 2b). Each category can be assigned to one or more of the following 4 senses—tactile, visual, auditory, olfactory—regarding gustatory stimuli; no stress-relieving devices were identified that incorporated taste (Box 2).

**Box 2:**
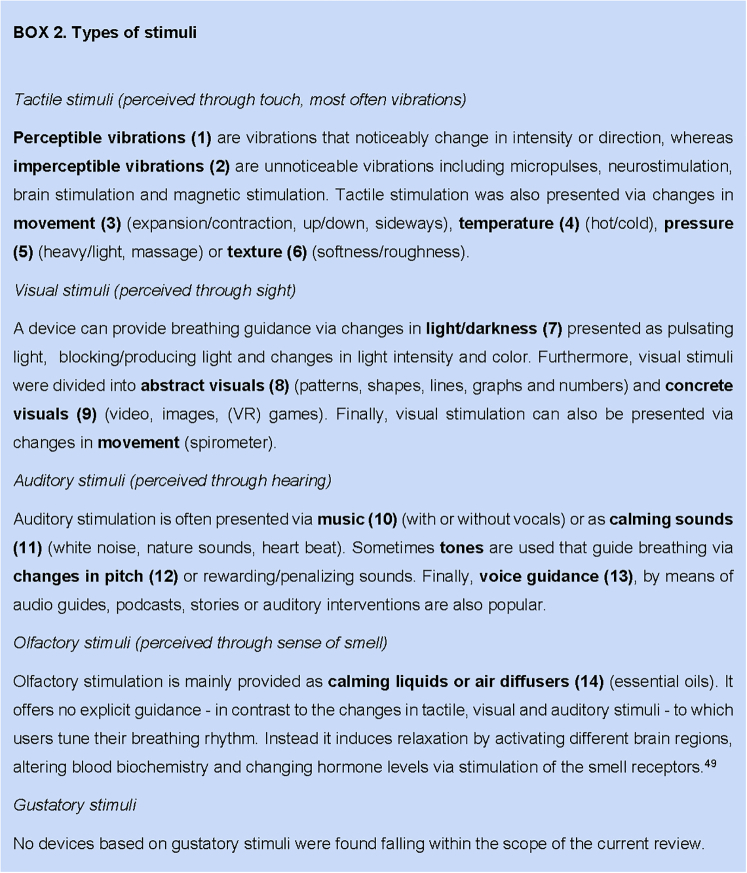
Types of stimuli.

### Evaluation of the Devices and Stimuli

In addition to identifying and categorizing the devices and their stimuli, we report on their feasibility, acceptability, usability, preferences, and effectiveness as described in the scientific literature. Nine different studies (randomized controlled trials and prospective studies) evaluated a wearable heart rate sensor that provided tactile (vibrations) and auditory feedback: the RESPeRATE. The results found that the device was not feasible to use in mindfulness training.^47^ However, results indicate that implementation of the device did result in high quality breathing synchronization with the device and a reduced breathing pace that improved clinical outcomes (blood pressure).^31^ The other studies found that its use reduced the acute and chronic effects of cardiovascular reactivity to mental stress,^54^ improved health-related quality of life,^54^ slowed down the progression of chronic heart failure, and^55^ in addition, the implementation of the device led to modest improvements in the frequency and severity of hot flashes^56^and urinary symptoms.^57^ However, these improvements were smaller compared with a music listening intervention. Finally, breathing with the device resulted in acute reductions in blood pressure and sympathetic activity^58^ and it was said to be a feasible and potentially promising nonpharmacological treatment adjunct for trauma^59^ and stress.^60^ Two experimental studies evaluated multimedia (video + audio) feedback on a smartphone, and 2 reported on using a smartwatch to provide breathing guidance and feedback.^49^^,^^52^^,^^53^ It found that the multimedia system improved meditation training effectiveness and experience, whereas the smartwatch presented reliable, real-time breathing measurements.

Another study reviewed a portable biofeedback device that provided visual feedback, that is, a graph, by an LCD screen.^62^ The device also awarded points for exhaling calmly. This device appeared to be a promising treatment adjunct for anxiety and was easily integrated into treatment. Four studies evaluated devices that used tactile breathing guidance: a vibrating car seat, a vibrating clip-on, a stuffed breathing toy, and a device with thermal feedback.^50^^,^^61^^,^^63^^,^^64^ Tactile guidance was often preferred over other types of stimuli because it was more natural to follow, less distracting, and easier to engage and disengage from. It also improved negative affect and reduced variations in mean heart rate and migraine. However, they did not find an effect on anxiety levels. Devices that incorporated auditory feedback, such as music, found positive results, lowering depression and anxiety.^63^ However, it was stated that preferred or accepted types of music can differ geographically, so cultural consideration might be required. Finally, 3 studies reported on the use of virtual reality (VR) in breathing guidance.^48^^,^^51^^,^^63^ Sometimes physical complaints were reported. However, different beneficial effects were associated with the use of VR: it improved emotion regulation and decision-making, promoted deep breathing, enhanced experiences, and encouraged user engagement. It also reduced stress, tension, fatigue, confusion, depression, anxiety, and other symptoms related to physical disorders. Especially in combination with biofeedback, the results were promising. However, VR appeared best suited for the young generation. It was concluded that user needs, experience, and preferences should be taken into account when designing a VR game.

## Discussion

Until today, little research has been conducted on the preferences of people toward stress reduction devices and their stimuli, apart from a few exploratory studies. However, more and more devices and associated stimuli (visual, auditory, tactile, or olfactory stimuli) that guide slow breathing and thereby reduce stress are brought to market. This study identified 46 types of devices incorporating 24 types of stimuli. They were organized into 7 device categories and 14 stimuli categories. The most frequent stimuli found were (i) concrete visuals such as numbers and graphs, presented using an application, software, or the device itself; (ii) light or darkness; (iii) sounds and tones; and (iv) vibrations. The fact that companies are largely investing in devices makes it important to understand people’s preferences toward them to steer development toward the preferences of the end-user and increase success rates. To do so, it is first necessary to create an overview of existing devices, their stimuli, and characteristics, which can then be used in subsequent patient-preference studies.

Current health technologies and products are typically developed top-down (eg, commercial strategy, previous innovations, and intuitive selection). This leads to people using products that do not adequately address their needs, which is certainly problematic in the area of mental health, an area that seeks to engage in highly personalized support.^67^, ^68^, ^69^, ^70^, ^71^, ^72^ There is a need to develop mental health products in a bottom-up manner (ie, taking into account the requirements, preferences, and needs of patients). Patient-preference studies are considered an evidence-based way to elicit patient preferences and are currently being used and recognized by regulators, developers, and health technology assessment or reimbursement agencies as important in the domain of drug and medical device development.^73^ Using these techniques in the consumer mental health sector is, however, innovative, and unique.

Because of the complex classification of and overlap between the results of this review, systematic categorization is not always evident. Categorization could be considered slightly random, with certain overlapping categories. However, we regard the chosen classification as useful to describe existing breathing devices and their incorporated sensory stimuli, and a first basis to inform subsequent preference studies that aim to understand the preferences of the end-user toward the different characteristics revealed in this review. This overview will, for instance, be used as the basis for a 2-year patient-preference study with stressed patients, including a large-scale survey, focus groups, and an in-lab assessment of the efficacy and preferences regarding meditation and breathing devices for stress reduction. It will also enable collaborative, patient-centered development of breathing devices for stress reduction. Furthermore, given the large number of commercially available products, a potential bias toward unpublished data on product development exists. We could only partially mitigate this bias by including not only commercially available products but also experimental devices not yet marketed to enhance the breadth of our analysis. This was achieved by incorporating both market analysis data and scientific research in our review. We believe this approach of combining commercial and academic sources provided a comprehensive overview of both established and emerging technologies in the field of stress-reducing breathing devices. Furthermore, it allowed us to capture a broad spectrum of user preferences, from those reflected in market trends to those discerned from experimental studies. In addition, most of the studies included were conducted independently, and some found negative results regarding the tested devices.

In the search for optimal solutions for people with stress, devices that support slow breathing have become popular and prevalent in the (mental) health care sector. These devices present breathing guidance by sensory stimulation using visual, auditory, tactile, or olfactory stimuli. Regarding the possible effects of different breathing devices, preliminary research found them to be a potentially promising nonpharmacological treatment for among others, insomnia, stress, trauma recovery, menopausal hot flashes, and high blood pressure.^57^, ^58^^,^^60^^,^^61^^,^^74^ The effectiveness and success of these devices, and end-user adherence toward these devices, however, strongly depend on how well they meet people’s preferences and needs. In fact, preliminary research indicates that people show a preference for specific types of devices and sensory stimuli in terms of eg, design, handling, methods, and effects (such as effect on stress levels and breathing pace). In addition, it is suggested that depending on the preference toward different devices and stimuli, they affect the physiological and psychological stress response in different ways.^28^^,^^35^

More specifically, studies on the effects of breathing exercises indicate that the way the breathing guidance is presented, influences its effects. Visual or auditory stimuli tend to be rather technical or cognitively demanding to follow.^28^ For example, when a breathing exercise was visually guided by a graph that indicated user progress, which required a high degree of concentration and attention, no relevant effects were found compared with a more relaxed breathing condition without visual guidance.^35^ The VR appeared to be a promising tool, however, this depended on user experience and age. In a preliminary preference study, an immersive breathing guidance system for relaxation was tested. Breathing guidance and feedback were provided using audio, tactile (vibrations), and visual (light) stimuli.^28^ Most users preferred the tactile feedback, even more in combination with synchronized audio. In addition, different intensities of tactile stimulation were preferred by different people. This means that allowing the personalization of stimuli can be a valuable asset. The visual stimulus was rated less important, probably owing to the tendency of users to close their eyes while breathing. Finally, in an evaluation study, researchers stated that, because breathing exercises require mental effort, which in turn might lead to new stress, the cognitive load should be lowered and relaxation should be increased.^28^ The tactile interface found most potential in inducing relaxation, on the basis of higher user satisfaction ratings. Therefore, the researchers hypothesized presenting breathing guidance using a tactile device (a hand-sized airbag) would be most effective and easy.

However, it may be difficult to distinguish the effects of specific stimuli because many breathing devices use a combination of different sensory stimuli. Also, it can be a challenge to determine (a golden combination of) the most relaxing stimuli, owing to differences in people’s preferences regarding sensory stimuli. Perhaps, people show preference for the sense that is most developed in their body. However, there is still much uncertainty about this. The first sense that develops in a person is touch.^75^ However, for a long time, it was thought that sight or hearing were the dominant senses.^76^ This accepted hierarchy of human senses—sight, hearing, touch, taste, and smell—eventually appeared not to be universal but strongly depended on culture, history, and experience.^76^ This could imply that specific senses can be more highly developed in a person or group and that individual preferences for different stimuli may differ depending on personal characteristics such as culture (preference heterogeneity). The link between the development of senses and preferences is however, under-researched, so this path needs further exploration.

In light of the preliminary research on preferences toward stress devices and their stimuli, in particular the preference for tactile guidance and feedback, this review clearly shows that a great deal of device developers nowadays do not take these preferences into account. It indicates an imbalance between breathing devices and the needs and preferences of their users. Device developers require such insights to stratify development into different device types that meet the needs of different types of people. The current overview of the existing breathing devices for stress and the sensory stimuli they use can be a first step in this process.

## Conclusion

As companies continue to invest heavily in the development of stress-reducing breathing devices, gaining a thorough understanding of users’ preferences toward these devices’ characteristics becomes paramount to optimize design, user experience, and success rates. To do so, this study provides an overview of 46 existing devices incorporating 24 different stimuli, which will be used to inform subsequent patient-preference studies that assess patient’s preferences toward these characteristics and inform the development of new devices for stress reduction.

## Potential Competing Interests

S.B. and E.H. are respectively co-founder and researcher at Moonbird B.V. The remaining authors declare that the research was conducted in the absence of any commercial or financial relationships that could be construed as a potential conflict of interest. The authors declare no competing interests.
