# Supplementary material for: Existing Meditation and Breathing Devices for Stress Reduction and Their Incorporated Stimuli: A Systematic Literature Review and Competition Analysis
Source: Mayo Clin Proc Digit Health. 2023 Aug 8;1(3):395–405. doi: 10.1016/j.mcpdig.2023.06.008 (PMC11975735; doi:10.1016/j.mcpdig.2023.06.008)
Supplement: Supplemental Data [file mmc1.pdf]

APPENDICES

Appendix 1

The results were obtained through a scientific literature study. Both Pubmed and Embase were used. Pubmed search strategy:

("Stress, Physiological"[Mesh:NoExp] OR “Physiological stress”[tiab] OR “Stress reduction*”[tiab] OR “Stress-reduction”*[tiab] OR “Stress Relief*”[tiab] OR "Meditation"[Mesh] OR "Relaxation"[Mesh] OR “Meditation*”[tiab] OR “Relaxation*” [tiab])
AND
("Respiration"[Mesh] OR "Inhalation"[Mesh] OR "Exhalation"[Mesh] OR “Respiration*”[tiab] OR “Inhalation*”[tiab] OR “Exhalation*”[tiab] OR “Breathing”[tiab])
AND
("Wearable Electronic Devices"[Mesh] OR “Device*”[tiab] OR “Medical device*”[tiab] OR “Device-guided breathing”[tiab] OR “Biofeedback system”[tiab] OR "Biofeedback, Psychology"[Mesh] OR “Respiratory guidance method*”[tiab] OR “Breathing guidance*”[tiab] OR “Breathing assistance system”[tiab] OR “Wearable”[tiab])
AND
(“Breathing exercise*”[tiab] OR “Treatment*”[tiab] OR "Wearable Electronic Devices"[Mesh] OR “Device*”[tiab] OR “Medical device*”[tiab] OR "Relaxation Therapy*"[Mesh] OR "Mind-Body Therapy*"[Mesh] OR “Relaxation Therapy”[tiab] OR “Mind-Body Therapy”[tiab])

209 results

Embase search strategy:

('physiological stress'/exp OR 'meditation'/exp OR 'physiological stress':ti,ab,kw OR 'meditation':ti,ab,kw OR 'stress reduction':ti,ab,kw OR 'stress-reduction':ti,ab,kw OR 'stress relief':ti,ab,kw)
AND
('breathing'/exp OR 'inhalation'/exp OR 'exhalation'/exp OR 'breathing exercise':ti,ab,kw OR 'breathing':ti,ab,kw OR 'inhalation':ti,ab,kw OR 'exhalation':ti,ab,kw)
AND
('devices'/exp OR 'medical device'/exp OR 'biofeedback'/exp OR 'wearable electronic devices':ti,ab,kw OR 'device':ti,ab,kw OR 'medical device':ti,ab,kw OR 'biofeedback system':ti,ab,kw OR 'respiratory guidance method':ti,ab,kw OR 'breathing guidance':ti,ab,kw OR 'breathing assistance system':ti,ab,kw OR 'wearable':ti,ab,kw)
AND
('relaxation training'/exp OR 'breathing exercise'/exp OR 'device therapy'/exp OR 'alternative medicine'/exp OR 'relaxation therapy':ti,ab,kw OR 'relaxation training':ti,ab,kw OR 'breathing exercise':ti,ab,kw OR 'device therapy':ti,ab,kw OR 'alternative medicine':ti,ab,kw)

158 results

Appendix 2a. Overview of meditation and breathing devices and stimuli from scientific literature and competition analysis

**Competition analysis**					
**Product name**	**Manufacturer**	**Function**	**Target group/goal**	**Type of device**	**Type of stimuli**
Muse	InteraXon	Multi-sensor meditation headband with real-time feedback on brain activity, HR, breathing, and body movements to build meditation practice.	Meditation	Headband	Visual feedback via app<br>Auditory feedback (sounds)
Core + app	Hyperice	Tactile meditation trainer (sphere) that<br>uses dynamic vibrations and feedback (HRV) to relax	Relaxation	Hand-held sphere	Tactile vibrations to guide relaxation via device<br>Visual feedback via app
Somnox	Somnox	Sleep robot to reduce stress and induce sleep via sounds and breathing movements	Stress<br>Sleep	Sleep robot	Tactile breathing movements from device to guide sleep<br>Soothing sounds from device
Dodow	Livlab	Relaxation/sleep device breathing guidance via light	Relaxation<br>Sleep	Projector	Visual (pulsing blue light) from device projections
emWave (+ software)	HeartMath	Portable device that measures stress parameters and coherence via ear or thumb sensor and gives biofeedback	Breathing guidance (heart coherence)	Portable sensor	Visual biofeedback via software
Inner Balance (+app)	HeartMath	Bluetooth sensor that measures stress parameters and coherence and gives biofeedback	Breathing guidance (heart coherence)	Portable sensor	Visual biofeedback via software

Smartwatch	Apple, Samsung, ...	Watch that records vital signs and provides guided breathing exercises via visuals or vibrations	Stress & activity<br>Sleep<br>Breathing guidance for relaxation	Smartwatch	Visual biofeedback via watch<br>Visual breathing guidance via watch<br>Tactile breathing guidance via watch (vibrations)
Activity trackers	Fitbit, Garmin, Polar, Beurer	Wearable monitoring and tracking fitness-related metrics with biofeedback	Stress & activity	Wearable activity tracker	Visual biofeedback via tracker
Spire stone	Spire health	Stress management & activity tracker (stone)	Stress & activity	Hand tension monitor (stone)	Visual biofeedback via app
Bellabeat accessories	Bellabeat	Activity, sleep, stress, meditation & reproductivity tracker as jewelry/accessories. Guided meditation/breathing exercises via the app	Activity, sleep, stress, meditation & reproductivity	Jewelry	Visual biofeedback via app
Oura ring	Oura Health Oy	Readiness, sleep and activity tracker (ring)	Sleep<br>Stress<br>Activity	Ring	Visual biofeedback via app
Somaticvision games	Somaticvision	Biofeedback gaming	Stress<br>Performance	Game	Visual biofeedback via video auditory biofeedback
b	breathe with b	Breathing hardware device (breather) for breathing training	Breathing training	Electrical breather	Vibrations, lights and sounds to guide via tool
Beurer stress releaZer +	Beurer	Tactile relaxation aid (pebble) and breathing trainer	Relaxation<br>Breathing guidance	Tactile pebble	Guidance via:<br><br>Tactile (low-frequency vibrations & heat function)

Calm Down app				App	auditory (music: forest/jungle/ocean)<br>Visual (illuminated ring that pulses)
Melo		Breathing stone/wearable	Wellness Mindfulness Anxiety	Handheld breath pacer/accessories	Tactile guidance (vibrations)<br>Visual guidance (counting via lights)
Noumic	Noumic	Hand tension monitor with biofeedback	Stress<br>Relaxation	Hand tension monitor	Visual feedback (lights: green/red)
iBreve	iBreve Limited	Wearable sensor technology for stress reduction with app feedback	Stress	Wearable sensor	Visual feedback via app
The Shift	Komuso Design	Physical breather for breathing training	Breathing training	Non-electric breather	No feedback or guidance<br>Physical breather
2breathe	Resperate Inc.<br>2breathe Technologies Ltd.	Sleep inducer sensor unit on a flexible belt + app to guide breathing via sound	Sleep/relaxation<br>Breathing guidance	Belt with sensor	auditory (sound guidance via app)<br>Visual feedback via app
Soft-tones	Resperate Inc.<br>2breathe Technologies Ltd.	Over-the-pillow high quality stereo speaker	Sleep/relaxation<br>Breathing guidance	Pillow/speaker tool	auditory guidance (via speaker)<br>Tactile (softness)
eVu-Senz TPS	Thought Technology	Portable sensor (thumb) registers data - app provides feedback +	Relaxation<br>Breathing guidance	Portable sensor	Visual feedback via app

(sensor) + app		breath pacer to increase relaxation		App	auditory feedback via app<br>auditory guidance via app (sounds, music, voice guide)<br>Visual guidance via app (game: change environment, play race)
GSR2 Biofeedback Relaxation System	Thought Technology	Galvanic skin resistance monitor. Measures stress responses of skin & sweat glands. As tension changes, pitch tone rises/falls	Stress<br>Relaxation	Skin resistance monitor	Auditory feedback: as tension increases, tone increases, as tension decreases, tone decreases
The Pip + apps	Pip	Thumb-held device measures stress levels via Electrodermal Activity with feedback via multiple apps	Stress	Thumb sensor	Visual feedback via app<br>Visual guidance via the app
Iom2	Integrated Listening Systems + Unyte	Biofeedback device with breathing and meditation guidance	Breathing guidance<br>Meditation	Portable measuring device	auditory guidance via device
Dreampad	Integrated Listening Systems + Unyte	Pillow with soothing music and gentle vibration to trigger relaxation	Sleep/relaxation	Pillow	Tactile guidance: vibrations (+ softness)<br>auditory guidance: music
Safe and Sound protocol	Integrated Listening Systems + Unyte	An auditory intervention to reduce stress and auditory sensitivity	Stress, anxiety	Auditory intervention	auditory guidance via headset

Sensate	Sensate (Bioself Technology)	Pebble with ultrasonic frequencies to reduce heart, breath and brain wave frequency	Stress, anxiety<br>Meditation	Tactile pebble	Auditory guidance: Ultrasonic frequencies<br>auditory guidance: sounds<br>Visual feedback via app
Versus headset + app	Neuro Management	Mobile EEG headset with Versus app to focus or reduce stress	Stress	Headset	Visual feedback via app
Dhyana + app	Avantari (Dhyana)	Wearable ring that tracks meditation via HRV biofeedback + app	Meditation	Ring	Visual feedback via app
Feelzing	Thync	Neurostimulation patch that stimulates SNS & PNS	Energy, focus and relaxation	Patch	Tactile neurostimulation<br>no guidance or feedback
Apollo Neuro + app	Apollo Neuroscience	Wearable that vibrates to balance NS	Balance/stress	Vibrating wearable (ankle/wrist)	Tactile guidance: vibrations<br>Visual guidance: via app<br>Visual feedback via app
Melomind + app	Melomind	Headset meditation tool that guides relaxation via alpha waves, biofeedback & app	Meditation	Headset	Tactile guidance: via alpha waves<br>Visual feedback via app
Dreamlight (Zen - Muse - Ease - Heat)	Dreamlight	Meditation sleep mask with light block, orange light, meditation sounds, music or heat	Meditation<br>Sleep<br>Relaxation	Sleep mask	Visual guidance: via orange light and light blocking<br>Tactile guidance via mask softness & heaviness + heat<br>auditory guidance via music
Neorhythm	Omnipemf	PEMF device with magnetic stimulation to target brain or body areas to induce sleep, relaxation and meditation	Sleep, relaxation and meditation	PEMF device	Tactile guidance via magnetic stimulation<br><br>No feedback

Calmigo	Calmigo	Mouthpiece with vibration, scent and feedback lights to calm breathing	Breathing guidance	Mouthpiece	Tactile guidance: vibration device<br>Smell guidance: calming scent<br>Visual guidance/feedback: lights on device
Hoomband	Livlab	Audio headband to fall asleep (meditations, noise, ASMR)		Headband	auditory guidance (stories, music, meditations)
Xen + app	Neuvana	Electrical signal via earbuds, targeting the vagus nerve in the ear.	Stress	Earbuds	
BreathBalanz	Breath In Balanz	Sleep inducer sensor unit on a flexible belt + app to guide breathing	Sleep/relaxation	Belt with sensor	Visual feedback via app
BE Buddy	Balancing elephants	The BE Buddy® is a multi-sensory breathing tool (tactile, visual) and comforting eye pillow to help people relax and relieve stress	Relaxation<br>Stress<br>Breathing guidance	Eye pillow	Visual (colours/figures/darkness) & tactile (soft & cherry pit & weight) guidance<br>Smell (fragrance)
(Weighted) Plush Animals		A pressure therapy technique via weighted plush animals to calm, relax, and relieve stress and breathing exercise (on belly)	Relaxation<br>Stress<br>Breathing guidance	Weighted plush animal	Visual (animals) & tactile (softness & weight) guidance
Hoberman sphere		Isokinetic structure: the movement of the sphere replicates the movement of the belly while breathing	Breathing guidance	Isokinetic toy	Visual and tactile (opening & closing) guidance via movement
Stress relieving		Tactile gadgets to relieve stress by distraction and tension release	Stress	Stress ball, pop bubble fidget	Tactile guidance (pushing & squeezing)

gadgets: Push pop bubble fidget & stress balls					
(Incentive) Respirometer & exerciser	Nuwik, Romsons, Wonder care	Spirometers that will help users exercise regularly and work on breathing	Breathing training	Spirometer	Visual feedback & guidance (movement of balls)
Calming liquid	Aromakids, Bach rescue spray, A Vogel..	Aromatherapy to relax	Relaxation	Aromatherapy	Sense of smell guidance
ZAZU Kids sound machine and projectors	ZAZU Kids	Animal-shaped sound machines and projectors to relax and guide users to sleep	Relaxation<br>Sleep	Sound machine	auditory guidance: soothing sounds, white noise, heartbeat.<br>Visual guidance: LED lights in 5 colors<br>Tactile guidance (soft animal)
Moonie	Moonie	Bunny cuddle that guides users to relax and sleep by reminding them of the environment in the womb, providing a sense of security.	Sleep<br>Relaxation	Plush animal	Visual guidance (night lamp)<br><br>auditory guidance (Soothing noises: heartbeat, water..)<br><br>Tactile guidance (softness)
Plush animal with breathing guidance	88 unlimited	Plush animal that guides breathing by belly movement	Breathing guidance	Plush animal	Visual guidance (movement animal)<br>Tactile guidance (softness)

Meditate Mate	Meditate Mate	Cuddly toy to sleep and relax with guided meditation and focus on breathing	Sleep/relaxation<br>Meditation<br>Breathing guidance	Plush animal	auditory guidance (meditation, music)<br><br>Tactile guidance (softness)
Moxie	Embodied	Companion robot to chat or play with. Also provides meditation guidance.	Meditation guidance<br>Breathing guidance	Companion robot	Visual, Tactile, auditory guidance and feedback
ChillFish	Tobias Sonne	breath-controlled biofeedback game for relaxation skills by monitoring the breathing via a feedback loop	Relaxation<br>Breathing guidance	Game/biofeedback device computer	Visual and auditory guidance and feedback via software
Breathing+ Breathing Race	Breathing Labs	Interactive breathing exercises gaming system controlled by the player's breathing using a specially designed headset. Breathing Toys, a line of electronic toys designed to engage people in breathing exercises. (Breathing Cars: competitive racing experience)	Breathing guidance	Game	Visual and auditory guidance and feedback
Zenimal	Zenimal	Portable, animal-shaped tool to develop a mindful meditation practice via guided audio meditations	Meditation	Speaker tool	auditory, tactile, visual guidance (music - texture - animal shape & colour)
Sleepcogni + app	Sleepcogni	Ring that measures stress parameters and provides biofeedback for relaxation and sleep	Stress<br>Sleep/relaxation	Ring	Visual, auditory and tactile cues via device and app

Mysa smart shirt	Mysa	smart shirt for relaxation by guiding breathing exercises using vibrotactile feedback	Relaxation<br>Breathing guidance	Smart shirt	Tactile feedback (vibrations + softness)
FOCUS + app	UCLouvain and Université de Lille	Handheld breath pacer with heart rate sensor to teach youngsters (6 to 16 years) to relax, understand their body and emotional functioning and to (re)take control of their stress via a defined breathing rhythm.	Stress<br>Breathing guidance	Handheld breath pacer	Guidance via change in light intensity and/or vibration of the device<br><br>Feedback via central gauge that fluctuates according to level of cardiac variability measured by the sensor
Kyto + app	Kyto fitness technology	Mobile heart rate and HRV monitor with ear clip and fingertip sensor<br>Also a version with a chest strap.	Breathing guidance<br>Heart coherence	Portable sensor	Visual feedback via (third party) apps (graphs)
Kyto smartband	Kyto fitness technology	Heart rate wristband that tracks HR & HRV while working out	Breathing guidance<br>Heart coherence	Smartwatch	Visual feedback via (third-party) apps
Zeez Sleep Pebble and Pad	Zeez	Pebble and pad for under your pillow to induce sleep	Sleep<br>Relaxation	Tactile pebble & pad	Tactile guidance (pulses via brainwave patterns)
DreemLab	Dreem	Headband for monitoring and automatic analysis of EEG and	Sleep	Headband	Visual feedback on app

		biosignals.<br>With accelerometer, audio measure and EEG sensor.			
Zen egg	Mikropis Holding	Wooden totem: observe subsided swaying and calm down. Massage with egg. Visual reminder to take a break.	Relaxation<br>Stress	Wooden totem	Visual guidance (movement of object)<br>Tactile guidance (massage)
Moment pebble + self-paced Moment program	Moment company	Pebble that enables you to take 30 second micro moments of mindfulness. With a gentle light that beats from within, it guides through short breathing exercises that help de-stress and re-energise.	Mindfulness<br>Stress<br>Breathing guidance	Pebble	Visual guidance (pulsing lights)
Philips SmartSleep Deep Sleep Headband	Philips	Wearable sleep headband and mobile app to improve the quality of sleep. Detects and monitors sleep (stages). Once in “slow wave sleep”, the algorithm triggers quiet audio tones to boost these slow waves, thus improving quality of sleep. An algorithm customizes the timing and volume of tones to your sleep pattern. The SleepMapper App will show you your sleep metrics and your sleep boost score.		Headband	Visual feedback via app<br><br>Auditory guidance (quiet audio tones)
Aidlab + app	Aidlab	Chest strap that tracks HRV Analysis, Activity Tracking, Temperature,	Sleeping<br>Breathing<br>Detection & monitoring	Chest strap	Visual feedback via app

		Heart Monitoring, and Respiration Rate			
Aidmed one	Aidlab	Chest strap that tracks HRV Analysis, Activity Tracking, Temperature, Heart Monitoring, and Respiration Rate, sleep sensor, cough detector	Sleeping<br>Breathing<br>Detection & monitoring	Chest strap	Visual feedback via app or software
Cardiomood bracelet (CardioWatch 287-1)	Cardiomood	Wireless remote monitoring via watch for collection of heart rate, heart rate variability, respiration rate, activity and sleep. Data is transmitted wirelessly from the device via the application or health cloud	Breathing<br>Activity & recovery	Smartwatch	Visual feedback via app or software
Airofit	Airofit	Mouthpiece that tracks your breathing, your smartphone gives you live feedback and guidance	Breathing	Mouthpiece	Visual feedback and guidance via app
**Scientific literature** (32, 65-83)					
**Product name/description +**	**Reference + date of search**	**Function**	**Target group/goal**	**Type of device**	**Stimuli**

**Manufacturer**					
InterCure Ltd RESPeRATE	32, 72-79<br>10/11/2021<br>21/11/2021<br>26/11/2021	Respiration sensor around the chest over clothing with a small computerized box for biofeedback	Stress reduction	Portable sensor	Auditory stimuli<br>two tone inhale-exhale musical melody
Stuffed Bear	83<br>21/11/2021	Stuffed plush animal	Relaxation	Plush animal	Movement of the stuffed animal's abdomen.
Helicor, Inc. Stress Eraser	71, 81<br>21/11/2021<br>26/11/2021	Biofeedback device<br>Measures HRV from the pulse in your fingertip via an infrared sensor and displays it as a wave to instruct users' respiration	Stress reduction, relaxation and relaxation training	Portable sensor (finger)	Auditory stimuli (sound)<br>Visual stimuli (wave)
Breath walk	67<br>10/11/2021	Auditory intervention synchronizing the footstep with appropriate breathing rhythm	Walking meditation	Auditory intervention	Visual and/or auditory mechanism
Heart Wizard ™ Stress Sweeper	82<br>21/11/2021	Biofeedback device<br>Ear-clip to earlobe or finger + USB pulse wave sensor in computer)	Anxiety reduction<br>(On internet: reduce stress, restore inner balance and improve overall health)	Portable sensor (finger/ear)	Visual and auditory stimuli<br>(pleasant pacing sound)
Biofeedback device which can sit comfortably on someone's hip	65<br>21/11/2021	Biofeedback device on hip that collects continuous respiration data and claims to provide real-data feedback when breathing patterns indicate tension	Stress reduction	Portable measuring device	Buzzing and vibration<br>Sending a message to the wearer's phone when it detects erratic, defined by fast and highly variable, breathing patterns<br>= visual feedback

Biopad	66<br>26/11/2021	Biofeedback gamepad<br>It monitors the players' physiology during gameplay, and manipulates signals from the game controller accordingly	Stress reduction	Game/biofeedback device computer	Biofeedback when the player's physiology deviates from the target state<br>Changes in the movements of the avatar/car
Driver's seat	68<br>26/11/2021	Driver's seat that guides the driver in proper breathing	Stress reduction<br>Improve mental health and overall wellness	Seat cover	Haptic stimuli vibrations (different patterns)<br>- the motors begin vibrating from the middle of the seat and then move outwards, like an accordion.<br>- vibration of rows going up and down to simulate counting up and down<br>- a spiral with vibrations emanating in spiraling circles<br>Voice guided stimuli<br>= simple instructions such as "inhale" or "exhale"
HearthMath emWAVE	32<br>26/11/2021	Small portable device using a single sensor (for HRV) to adapt the breathing guidance rate based on the current state of the user	Stress reduction and relaxation	Portable sensor	Visual and auditory stimuli (colored LEDs)
Rendever Headset virtual reality	69<br>26/11/2021	Headset	Stress reduction and anxiety reduction (in rehabilitation context)	Headset	Virtual outdoor environment (a meadow on a mountain top overlooking a mountain range)<br>Soft music plays in the background<br><br>Visual stimuli breath bubble in front of them that grew lager and became smaller

Spire Health Spire Stone	80 26/11/2021	Monitoring and feedback device<br>Clip-on wearable<br>Worn on the waistband or the center or side of a bra	Reduce anxiety, negative effect and stress	Portable sensor (clip)	Tense alerts via the device (vibration)<br>Push notification via the app = visual feedback/guidance
Touch Blanket	32 26/11/2021	Breathe with the ocean concept<br>Blanket and headphone	Relaxation	Headset + blanket	Haptic stimuli (haptic waves synchronized with audio samples of approaching and retreating ocean waves on a shoreline)<br>Visual stimuli (LivingColors lamps with yellow/orange to imitate the colors of sand and sun and blue to imitate the color of the sky or ocean)
SOLAR	71 26/11/2021	Virtual reality game	Stress-reduction	Game	Visual stimuli<br>- Color of meditation circle (meditation score)<br>- Circle getting smaller and bigger (as the user exhales and inhales)
Bellabeat LEAF	70 26/11/2021	Motion sensor to detect body movement associated with breathing (attach the device to chest or abdomen)<br><br>A wellness tracker keeping up with your lifestyle and your wellness goals<br><br>Wear it as a bracelet, necklace or as a clip	Mediation and better sleep habits	Jewelry/wearable sensor	App feedback (visual stimuli)
Zephyr Technology Zephyr HXM	70 26/11/2021	Chest strap that monitors respiration	/	Chest strap	App feedback (visual stimuli)

MindfulWatch	70, 71<br>26/11/2021	Smartwatch that monitors respiration during meditation by detecting subtle wrist motions	Meditation	Smartwatch	Feedback via watch (future work)
Spire Health<br>Spire Health Tag	80<br>26/11/2021	Monitoring respiration<br>Adheres semipermanently to clothing (clip)<br>+ gives feedback notifications	Reduce pain, anxiety and stress (context of chronic pain in adult survivors of childhood cancer)	Portable sensor (clip)	App sends u notifications if you're stressed<br>(visual, haptic and auditory stimuli)
Hexoskin	70<br>26/11/2021	The Hexoskin Smart Garments include textile sensors embedded into comfortable garments for precise and continuous cardiac, respiratory, and activity monitoring.	Control your breathing, increase your performance and reduce stress	Smart shirt	App feedback (visual stimuli)

Appendix 2b. Unique types of devices and stimuli (without duplicates)

Type of device	Type of guiding stimuli	Type of feedback stimuli
N = 46	N = 24	
**Attachable to the head:**<br>headband<br>PEMF device<br>headset<br>earbuds<br><br>**Huggable devices**:<br>sleep robot-pillow<br>weighted plush animal<br>plush animal<br>pillow<br>Tactile pebble<br>Blanket<br>Seat cover<br><br>**Eye masks:**<br>sleep mask<br>eye pillow<br><br>**Handheld:**<br>handheld breath pacer<br>stone/wooden totem<br>hand tension monitor (stone)<br>skin resistance monitor (hand)<br>hand-held sphere<br><br>**Mouth controlled devices:**<br>mouthpiece<br>electrical breather<br>non-electrical breather<br><br>**Toys:**	**Tactile**<br><br>*Perceptible vibrations*<br><br>*Imperceptible vibrations*<br>ultrasonic frequencies<br>neurostimulation: micropulses/magnetic stimulation/alpha waves<br><br>*Movement*:<br>expanding-contracting<br>push & squeeze<br><br>*Temperature*<br><br>*Pressure*:<br>weight/massage<br><br>*Texture*:<br>softness, stimulation<br><br>**Visual**<br><br>*Movement:*<br>expanding-contracting<br>movement of objects<br><br>*Light/dark:*<br>light (pulsations, intensity changes, on/off)<br>darkness<br>coloured light (blue, orange)	**Tactile**<br>*Perceptible vibrations*<br><br>**Visual**<br><br>*Movement:*<br>visual feedback via movement<br><br>*Light/dark:*<br>visual feedback via lights (colour change)<br><br>*Abstract & concrete visuals:*<br>visual feedback via app (graphs - parameters - visuals)<br>visual feedback via software (video - graphs - parameters)<br>visual feedback via device itself (graphs - parameters - visuals - fluctuation gauge)<br><br>**Auditory**<br><br>Tones:<br>*changing pitch, rewarding sounds*<br><br>**No feedback**

isokinetic toy<br>stress ball<br>pop bubble fidget<br>spirometer<br><br>**Wearables:**<br>jewelry/accessories<br>ring<br>smartwatch<br>wearable activity tracker<br>belt with sensor<br>chest strap<br>thumb sensor<br>patch<br>vibrating wearable<br>smart shirt<br>portable measuring device<br>portable sensor<br><br>**Stimuli-related products**<br><br>*Visual product*<br>application<br>game<br>biofeedback device computer<br>projector<br><br>*Auditory product*<br>auditory intervention<br>sound machine<br>speaker tool<br><br>*Aromatic product*<br>aromatherapy<br><br>*Combination of stimuli*<br>companion robot	*Abstract visuals:*<br>guiding visuals (pulsations, lines, changing environment)<br><br>*Concrete visuals:*<br>gamification/video<br><br>**Auditory**<br>*Music (with/without vocals)*<br><br>*Calming sounds:*<br>white noise, nature sounds, heartbeat<br><br>Tones:<br>changing pitch, rewarding sounds<br><br>*Voice guidance:*<br>intervention, podcast<br><br>**Olfactory**<br><br>*Aromas:*<br>calming scent<br><br>**No guidance**	
